# Co-Adaptation of Physical Attributes of the Mammalian Female Reproductive Tract and Sperm to Facilitate Fertilization

**DOI:** 10.3390/cells10061297

**Published:** 2021-05-24

**Authors:** Chih-Kuan Tung, Susan S. Suarez

**Affiliations:** 1 Department of Physics, North Carolina A&T State University, Greensboro, NC 27411, USA; 2 Department of Biomedical Sciences, Cornell University, Ithaca, NY 14853, USA; sss7@cornell.edu

**Keywords:** sperm, vagina, cervix, uterus, oviduct, fertilization

## Abstract

The functions of the female reproductive tract not only encompass sperm migration, storage, and fertilization, but also support the transport and development of the fertilized egg through to the birth of offspring. Further, because the tract is open to the external environment, it must also provide protection against invasive pathogens. In biophysics, sperm are considered “pusher microswimmers”, because they are propelled by pushing fluid behind them. This type of swimming by motile microorganisms promotes the tendency to swim along walls and upstream in gentle fluid flows. Thus, the architecture of the walls of the female tract, and the gentle flows created by cilia, can guide sperm migration. The viscoelasticity of the fluids in the tract, such as mucus secretions, also promotes the cooperative swimming of sperm that can improve fertilization success; at the same time, the mucus can also impede the invasion of pathogens. This review is focused on how the mammalian female reproductive tract and sperm interact physically to facilitate the movement of sperm to the site of fertilization. Knowledge of female/sperm interactions can not only explain how the female tract can physically guide sperm to the fertilization site, but can also be applied for the improvement of in vitro fertilization devices.

## 1. Introduction

Much has been written about the challenges confronting mammalian sperm soon after they are deposited in the female reproductive tract, including: the long distance to the oocyte, the minuteness of the oocyte as a target, the bends and folds in the tract walls [1,2,3], thick mucus in parts of the tract [4], fluid flows outward toward the vagina [5], vaginal acidity [6] and immune responses to sperm [7]. All but the last two of these challenges are physical in nature.

These challenges may have originated, at least in part, to protect the female from invasive pathogens. In addition, the challenges are thought by many to serve to select the “best” sperm (strongest, fastest, most competent to fertilize) [3]. More than that, however, these challenges are also attributes that result from the co-evolution of the female reproductive tract and sperm to support fertilization [8]. As attributes, they may provide some guidance to steer sperm toward oocytes.

In this review, we will focus on how the physical attributes of the female tract (walls, fluid movement, and viscoelasticity) and of the sperm (shape, elasticity and rigidity) have apparently co-evolved to promote sperm movement to the site of fertilization. We will begin with an overview of the structures of mammalian sperm and the female reproductive tract to provide a basic background for the discussion of their physical interactions.

## 2. Physical Structures of Sperm That Affect Response to the Female Physical Environment

**Figure 1:**
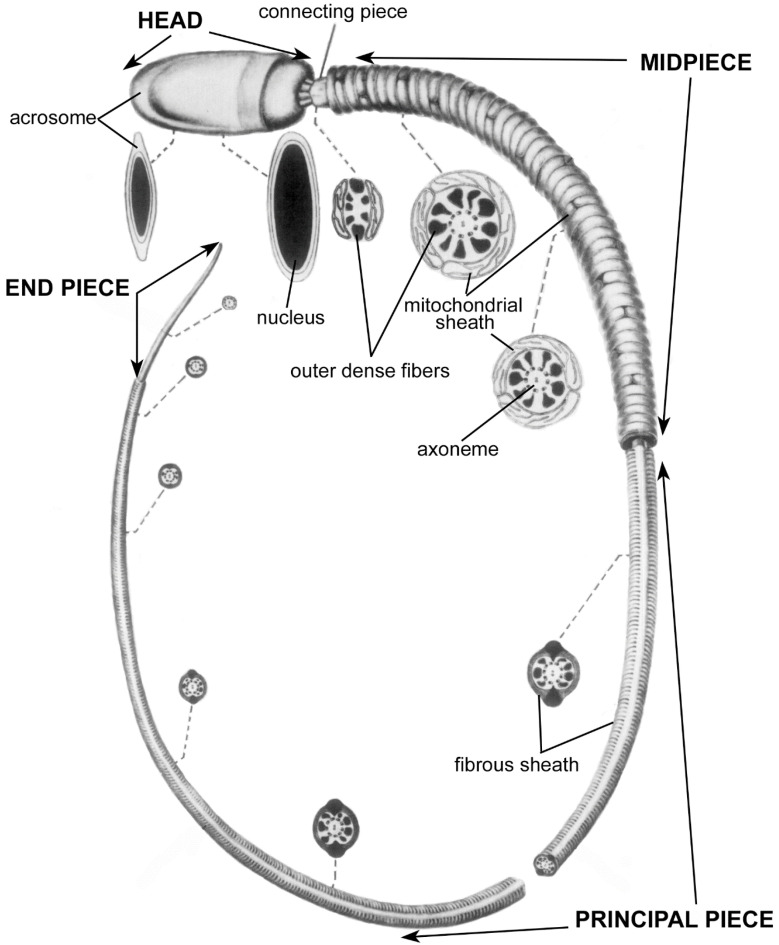
Basic anatomy of mammalian sperm, used with permission from Ref. [9]. Copyright 1975 Elsevier. In this drawing, the plasma membrane has been omitted in order to reveal internal structures. Cross sections are shown at various points along the length of the sperm, indicated by dashed lines. Mean lengths of sperm of mammalian species vary from 33.5 µm to 356.3 µm; the length of the head is less variable than the length of the flagellum, and averages roughly 8 µm [10].

The anatomy of the mammalian sperm head and flagellum is depicted in Figure 1, which shows structures that have been revealed by the removal of the plasma membrane and a thin layer of cytoplasm. For detailed descriptions of sperm anatomy, see Ref. [9].

The main components of the sperm head are the cell nucleus and the acrosome. This nucleus is highly compacted and contributes to the rigidity of the sperm head. The head rigidity is further strengthened by a dense layer of cytoskeletal proteins outside of the nucleus called the perinuclear theca [11]. The heads of most mammalian sperm are flattened ovoids; the ovoids of the heads of several species of rodents are interrupted by hooks [12]. The anterior portion of the nucleus is capped by the acrosome, a compact, membrane-enclosed organelle that contains hydrolytic enzymes. The acrosomal enzymes play various roles in preparing the sperm for fertilization and in the fertilization process itself (for more information, see Refs. [13,14]).

The sperm flagellum (tail) is anchored firmly to the head by the connecting piece and is divided into three regions: midpiece, principal piece, and end piece. The flagellum consists primarily of dynein molecular motors, skeletal elements, and mitochondria (Figure 1).

The dynein molecular motor proteins are anchored at the axoneme, which is a highly conserved microtubule-based skeletal structure in the core of the flagellum. The skeleton of microtubules and associated proteins provides both some rigidity and some elasticity to the flagellum [15]. The dynein proteins hydrolyze ATP to generate energy, which is used to form bends along the flagellum (for more information see Ref. [16]). Due to the spreading of bend formation down the flagellum, and to elastic responses to bending, a waveform propagates down to the end of the flagellum, thereby pushing the sperm forward [17] (for more information see Ref. [18]). In forward-moving sperm, the head can be seen to rotate about its longitudinal axis, which is called rolling, or the head can essentially remain within a plane, which is called planar movement [19]. High speed images of swimming mammalian sperm indicate that the two swimming patterns reflect subtle complex differences in flagellar beating patterns that are not yet well understood [20,21,22,23].

Whereas the axoneme forms the skeletal core of the entire flagellum, two additional skeletal elements surround the axoneme in parts of the flagellum: the outer dense fibers and the fibrous sheath. There are nine outer dense fibers, which surround the axoneme in the midpiece and principal piece (Figure 1). These fibers increase the tensile strength of the flagellum [24], and are thought to protect it from the fluid shear that sperm encounter during ejaculation, as well as from strong currents produced by muscular contractions in the female reproductive tract. The outer dense fibers are strongly anchored in the connecting piece to maintain the connection of the flagellum to the head. By anchoring the flagellum to the head, the connecting piece transmits shearing forces generated by the flagellar bending to the head [25]. Several roles of the connecting piece in motility have been proposed [26,27], yet detailed evidence is still emerging.

The fibrous sheath surrounds the outer dense fibers in the principal piece of the flagellum (Figure 1). It is thought to provide additional mechanical strength and elasticity to the flagellum; however, it also anchors enzymes involved in signaling and metabolic pathways in the principal piece [28,29].

Lastly, a string of tubular-shaped mitochondria connected end-to-end is wrapped in a tight helix that surrounds the outer dense fibers in the flagellar midpiece (Figure 1).

The shapes, organization, rigidity, tensile strength, and elasticity of the structures described above affect how sperm react to the physical attributes of the female reproductive tract they encounter; specifically, how these encounters affect the speed and direction of sperm swimming.

## 3. Physical Aspects of Female Environment That Affect Direction and Speed of Sperm Movement

### 3.1. Overview of Mammalian Female Reproductive Tract

**Figure 2:**
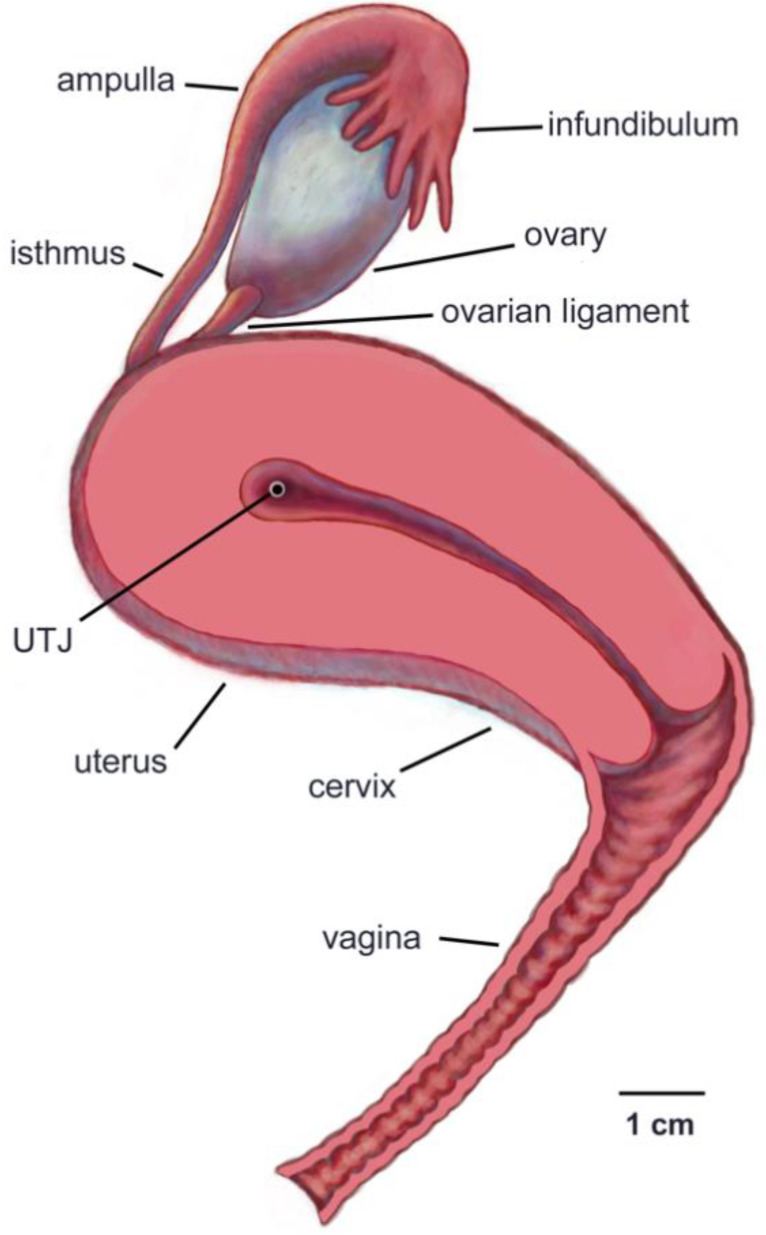
Left lateral view of the human female reproductive tract. The uterus, cervix and vagina have been bisected to show the entrance to the cervix and the uterotubal junction (UTJ). Only the right ovary and oviduct are shown. The ovarian ligament attaches the ovary to the uterus.

**Figure 3:**
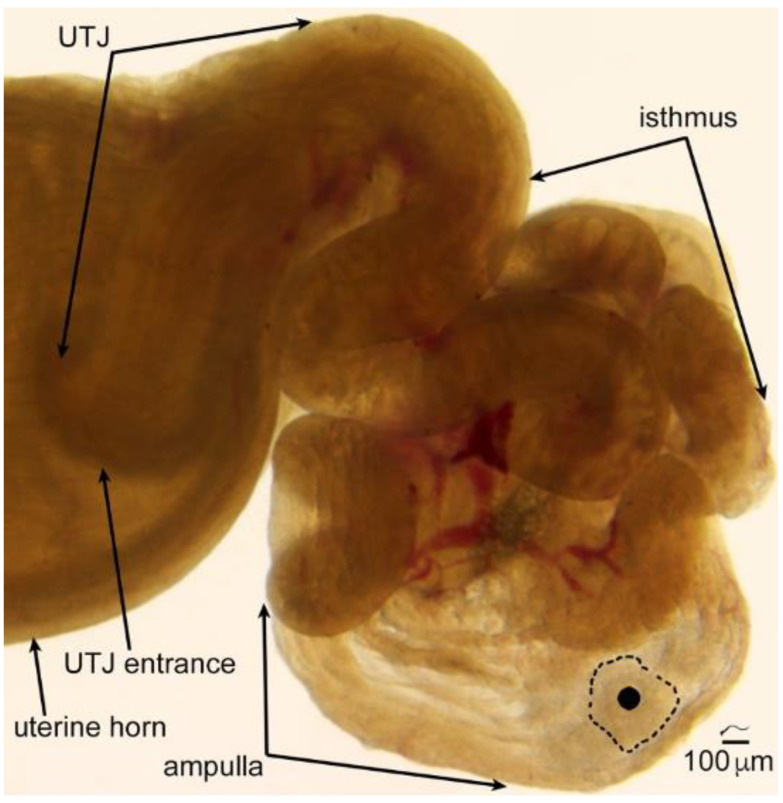
A transilluminated mouse oviduct. The ovary has been removed, but the coiling of the oviduct has been left in place. The size of mouse sperm (about 125 μm) is illustrated above the scale bar. To appreciate the size of the oocytes and cumulus in the ampulla, one of the oocytes has been covered by a solid black circle and the border of its cumulus indicated by a dotted line. Note that longitudinal folds line the uterotubal junction (UTJ) and the ampulla, while transversely oriented folds that form pockets can be seen in the isthmus, the site of sperm storage. Only the tip of a uterine horn is shown. Used with permission from Ref. [38]. Copyright 2010 Oxford University Press.

The anatomy of the female reproductive tracts of representative mammalian species (human, mice, cows) are shown in Figure 2 and Figure 3 and Figure 8b. The mammalian female reproductive tract is a chain of sequentially connected tubular organs that divides into two chains at a species characteristic point. The basic functions of the tract include sperm migration, storage, and fertilization. The tract also supports the transport of oocytes and development from the fertilized egg stage through to the birth of offspring. Further, because the tract is open to the external environment, it must also provide protection against the invasion of pathogens. The following overview is focused on the structural aspects of the tract that are critical for sperm migration/transport and storage. In this and other sections, the term “up” will be used to indicate “toward the ovary”, and “down” to indicate “toward the vagina”.

The vagina is basically an elastic tube. It is the site for semen deposition in some species, such as cows (*Bos taurus*) and humans, but is bypassed in other species, such as pigs (*Sus scrofa*) [30]. Regardless, sperm that are destined to migrate up the tract spend little time in the vagina, because the acidity of vaginal fluid, which serves to kill bacterial pathogens, can also kill sperm [31,32].

The cervix regulates the passage of sperm into the uterus, either directly, as in species which deposit semen in the vagina, or via interacting with the penis, as in species that bypass the vagina during insemination. It has thick walls lined by mucosal folds and covered by ciliated cells [5] and secretory cells, whose major product is mucus [33].

The uterus, which is the site of implantation and development of embryos, contains a muscular wall capable of producing strong, coordinated contractions that play roles both in transporting sperm up the tract and in birthing offspring. Primates have a single uterus, due to the developmental fusion of paired embryonic Müllerian ducts: however, in most other mammals the embryonic ducts fuse only partially or not at all, leading two long tubular structures called uterine horns [34].

The oviducts (also known as fallopian tubes), consist of four regions: uterotubal junction (UTJ), isthmus, ampulla, and infundibulum [35].

The UTJ regulates the passage of sperm from the uterus into the oviducts, and the passage of early embryos out of the oviducts into the uterus. The inner surfaces are lined by tissue folds that are more elaborate in some species than in others.

The isthmus of the oviduct is the site in which sperm are stored before the egg is released into the oviduct. The length of sperm storage in mammals ranges from about a day in mice and cattle [36] to months of winter hibernation in some species of bats [37]. The inner surface in most mammals is lined by folds that form pockets for sperm storage (Figure 3).

The ampulla of the oviduct serves as the site of fertilization and early embryo development. Whereas the tissue folds lining the inner surface of the isthmus tend to orient transversely to form pockets for sperm storage, the ampullary folds are oriented longitudinally (Figure 3 and Ref. [2]).

The infundibulum opens out toward the ovary. It is lined by cilia whose primary function is to transport ovulated eggs from the ovary into the oviduct. In eutherian mammals, the oocytes that enter the oviduct are surrounded by cumulus cells, a gel-like matrix, and the proteinaceous zona pellucida [39].

In the next section, we discuss the basic physical properties of the tubular organs of the female reproductive system that affect the movement of sperm.

### 3.2. Walls

It has been known for more than half a century that, instead of being distributed uniformly across a fluid-filled space, sperm are overwhelmingly found swimming close to liquid−solid interfaces [40] that hereafter will be referred to as walls. This accumulation of sperm at walls is primarily driven by the fact that sperm are “pusher microswimmers” [41]. Pusher microswimmers are cells or organisms that are 2–1000 µm in length, and are propelled by pushing fluid behind them. Sperm from various mammalian species range in length from about 30–350 µm [10]. Unlike larger swimmers, such as humans or fish, the mass of the microswimmer is small and the kinetic energy of its swimming becomes rapidly dissipated by the viscosity of the surrounding fluid if it stops swimming [42]. That is, viscous forces dominate over inertial forces, such that a simple watery environment acts as a thick syrup to microswimmers, and they generate negligible turbulence in the fluid that flows around them. It has been shown that all pusher microswimmers (sperm and otherwise) generate similar fluid flow patterns when swimming [43,44].

When pusher microswimmers approach a wall, due to their geometry, their heads collide with the wall and lead to the accumulation of microswimmers at the wall [45]. Further, as microswimmers get closer to a wall, the flow generated by their swimming is altered because there is no flow at the wall; this is referred to as a no-slip boundary condition. The hydrodynamic effect of the wall leads to a flow that further attracts microswimmers to, and prevents them from turning away from, the wall [46,47]. This passive physical mechanism combining collision and hydrodynamics leads to the accumulation of sperm at walls.

It has been estimated that the hydrodynamic attraction of sperm is greatest within 6 µm of a wall [43], which is roughly the length of a mammalian sperm head. At this point, sperm turn and start swimming along the wall. It is more common than not for them to remain swimming along it. Depending on the flagellar beating pattern, the sperm can remain swimming as close as 1 µm from the wall [48].

**Figure 4:**
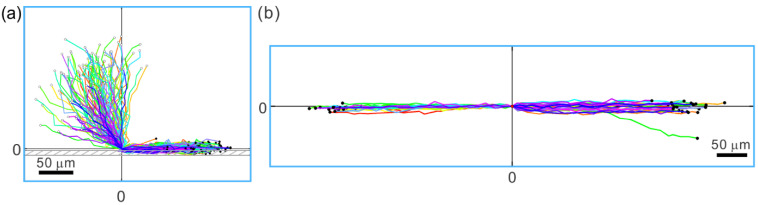
Tracks of sperm interactions with solid structures, as seen looking down from above. (**a**) Sperm that approach a sidewall from all angles (starting points denoted with open dots) swim along the sidewall (end points denoted with filled black dots) after hitting the wall. The location of wall contact is adjusted to (0,0) for each trajectory. (**b**) When sperm encounter a microgroove structure, the overwhelming majority of them remain in the groove. The starting points of the tracks are adjusted to (0,0). End points of the sperm trajectories are indicated by black dots. Note that sperm swim in either direction from the point of entry into the microgroove. Only one out of 50 sperm swam out of the microgroove. Adapted from Ref. [53]. Copyright 2015 by the authors.

The tendency of sperm to swim along walls has profound implications for directing sperm movement. As sperm are more likely to swim next to a wall than in the middle of a fluid space, they are even more likely to swim along the corner formed by the meeting of two walls than along a two-dimensional (flat) surface (see Figure 4a) [49,50,51]. Consequently, corners provide an effective guidance mechanism for sperm migration. Further, when sperm enter a groove-like structure with the width of the groove not much larger than the width of the sperm head, they predominantly remain swimming in the groove (see Figure 4b) [52,53]. This last point is particularly pertinent to sperm migration in the female reproductive tract, since “microgrooves” of the appropriate size are found in the walls of parts of the female tract; for example, in the walls that line the passageway of the bovine cervix [5].

### 3.3. Fluid Flow

As first observed more than six decades ago [54], sperm orient their swimming against a gentle fluid flow; specifically, a flow that is too weak to sweep sperm downstream. Recently, this phenomenon has received renewed interest, resulting in elucidation of the mechanisms that create sperm upstream swimming, known as rheotaxis. The rheotaxis of sperm has been shown to result from a physical, hydrodynamics-based, passive mechanism (i.e., it does not require active signaling from the sperm [55]) as follows [56,57]. Fluid flow is primarily generated in the female reproductive tract by ciliary action and muscle contraction, [1]. The flow created by these processes naturally forms a velocity gradient near the walls of the tract as mentioned above. Given that the sperm head is larger than the tail, and that sperm tend to swim near walls, the head generally experiences more hydrodynamic resistance (drag) than the tail. When there is a flow that moves the sperm downstream along a solid wall, the head experiences more resistance in the direction opposite to the flow direction, causing the sperm to swing around to orient against the flow. Once sperm are oriented to swim upstream, their active swimming moves them against the flow [56,57]. Over a range of flagellar beating amplitude and asymmetry, including increases induced by a rise in intracellular Ca^2+^, sperm behave similarly when orienting into a flow [58].

**Figure 5:**
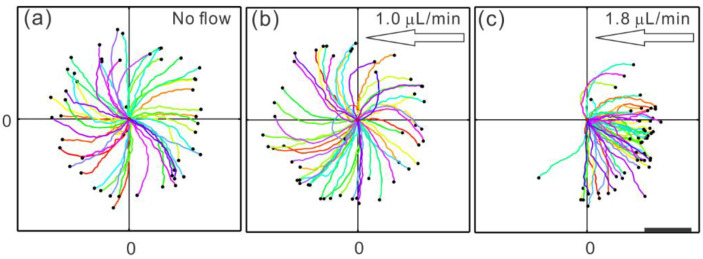
Bull sperm response to fluid flow. The coordinate (0,0) marks the starting point of all of the tracks, which were followed for 2.8 s. (**a**) When there is no flow, sperm exhibit curved trajectories as a segment of their near-circular tracks. (**b**) When the flow rate is below the threshold for upstream swimming (which was measured as 1.1 µL/min), sperm trajectories drift downstream, i.e., the trajectories to the left are extended further and curved less than the ones to the right. (**c**) Once the flow rate is above the onset, sperm exhibit nearly linear trajectories with an upstream component. (a) and (c) adapted from Ref. [57]. Copyright 2015 American Physical Society. (b) adapted from Ref. [53]. Copyright 2015 by the authors.

Sperm and other pusher microswimmers typically exhibit nearly circular trajectories when swimming on a flat surface [59,60]. This circular swimming plays a role in determining the window of flow speeds in which upstream swimming occurs. Some of this is attributed to the rotation of sperm along the long (head-to-tail) axis [61], and some of it is a result of asymmetric flagellar beating [62]. The circular trajectory prevents upstream swimming in a flow rate below a certain threshold, which is determined by how quickly the sperm completes a circle and how strong the hydrodynamic resistance (or drag) is between the sperm head and the wall [57]. The effects of fluid flow rates below and above the threshold can be seen in Figure 5. If the flow rate is below the threshold, the sperm remains swimming in its circular trajectory, although the circles drift downstream (Figure 5b). Once the flow rate exceeds the threshold, the sperm trajectory becomes linear (Figure 5c). The linear trajectory has a component of orientation in the upstream direction, and the angle from the exact upstream orientation decreases as the flow increases. Since the sperm is now oriented somewhat against the flow, as long as the sperm swims faster than the flow speed, it remains engaged in upstream swimming. However, if the flow speed becomes much faster than the fastest possible sperm swimming speed, even if the sperm is oriented perfectly against the flow, it will be swept downstream relative to the surface. Since sperm occasionally fluctuate away from a wall, where the flow is a lot stronger, they can be caught up by a flow and carried downstream.

All pusher microswimmers, including *Escherichia coli* bacteria, swim near a wall and have a larger head than tail, and thus can exhibit rheotaxis. *E coli* are normally found in the vagina, but not in the uteri, of healthy cows [63]. Could motile *E coli* be led up the bovine female reproductive tract by the pro-vaginal fluid flow in the tract?

Using the formulas in Ref. [57] we estimated that *E coli* require a flow rate double that of bull sperm in order to break their circular trajectory to orient upstream (using data from Ref. [60]); however, *E coli* swim at a speed of only 10–20 µm/s [60,64] as compared to 120 µm/s for bull sperm [57]. As a result, the range of distance between *E. coli* and the wall that will allow them to swim against a flow is a lot smaller than the range for sperm [65] and so it is far less likely that an *E coli* would ascend the tract than would a sperm. This observation possibly represents an example of co-evolution of female and male traits to select for sperm migration and restrict the migration of pathogens (see Section 5).

Given that sperm of different species have different head shapes and tail lengths, the flow rates that facilitate sperm upstream swimming would be different from species to species, and it would be interesting to examine how the natural flow rate in the female reproductive system is tuned to the window of flow rates that induce rheotaxis in sperm.

Ciliary action is the main source of the gentle fluid flows in the mammalian female reproductive tract and is known to create a downward flow in the oviduct of various species [66], including humans [67]. It has also been found to play a role in creating pro-vaginal flow in the bovine cervix [5]. These gentle downward flows could assist sperm in swimming up the tract toward the site of fertilization.

In mice, periovulatory pro-ovarian smooth muscle contractions occur in the isthmus (lower portion) of the oviduct [68] and carry sperm in a back-and-forth motion toward the fertilization site [69]. In contrast, in rabbits, mating immediately results in strong pro-ovarian peristaltic muscle contractions that are temporarily generated throughout most of the female tract. The contractions produce strong pulsatile flows that carry some of the sperm up the tract [70]. However, the flow generated by the contractions is apparently so strong that it kills the sperm that are rapidly transported as far as the oviduct, such that it is unlikely that these sperm ever fertilize oocytes [70]. A function for this rapid transport has not been established, although it has been proposed that the dead sperm serve to signal the tract to prepare for fertilization [70].

### 3.4. Fluid Viscoelasticity

In vivo, sperm often swim in viscoelastic fluid [71] (for example, the rheology measurement of estrous bovine cervical mucus can be found in Ref. [53]). Viscoelasticity, a property that often occurs in complex polymeric solutions, is a combination of both viscosity (thickness) and elasticity. Viscosity is a typical liquid property: as liquid flows, viscosity dissipates energy and impedes fluid movement. Elasticity, on the other hand, is more of a property of a solid, in that the substance has a particular shape, and when it is deformed, there is energy stored within the material to restore its original shape. A simple liquid does not have intrinsic shape; rather, the shape of the container determines the shape of the liquid [72]. In complex viscoelastic fluids that contain long-chain flexible polymers, the entanglement of the polymers provides a scaffold-like structure that maintains a shape at short time scales [73]. However, since the polymer molecules in liquids are not cross-linked with each other as they are in gels, they can slide relative to each other; consequently, a flow is observed at longer time scales. In typical media, such as those used in labs (and most IVF clinics), sperm swim very differently from how they swim in viscoelastic media.

One approach for experimentally developing our understanding of the effects of viscoelasticity on sperm movement is to separate the effects of viscosity and elasticity. For example, the polymer polyvinylpyrrolidone (PVP) can be used to increase the viscosity without adding significant elasticity to the fluid, thereby allowing the fluid to remain Newtonian viscous (i.e., maintaining the same viscosity across different shear rates) [74,75]. By increasing fluid viscosity with PVP, it was shown that sperm swim slower as viscosity increases [74] (the relation between PVP concentration and viscosity can be found in Ref. [76]). Assuming that sperm flagellar beating generates the same amount of stress in all fluids, sperm will naturally move less (more slowly) in a more viscous fluid. Indeed, PVP is commonly used in intracytoplasmic sperm injection (ICSI) to slow down a sperm enough for a technician to capture it for injection into an oocyte [77]. A more interesting observation is that, when sperm are swimming near a wall, as the fluid viscosity increases, the sperm change from a predominantly rolling motility to a planar beating pattern [78,79]. Rolling and planar beating sperm are not fundamentally two different phenotypes—if a planar beating sperm departs from a wall, the same sperm will be seen rolling again [48].

It would have been nice if there existed a material that could be added to sperm medium to increase only the elasticity of the fluid. Unfortunately, the polymers that give the fluid elasticity also increase its viscosity. Two polymers are often used to add viscoelasticity to sperm medium: methylcellulose (MC) and long-chain polyacrylamide (PAM). Within a concentration range, an MC solution can be modeled using the linear viscoelastic Maxwell fluid model [80,81], which is weakly elastic. Due to its weak elasticity, MC is sometimes used primarily for increasing viscosity [48,82,83]. Similar to increasing viscosity using PVP, in MC solution, sperm flagellar beating becomes dominated by planar beating when sperm swim close to a wall [48,82]. Despite the weak elasticity of MC solutions, it has been shown that sperm swim faster in an MC solution with slightly higher viscosity than in a PVP solution with a slightly lower viscosity [74], suggesting that the elasticity of the MC solution allows sperm to swim faster. Indeed, sperm swim significantly faster in a highly elastic PAM solution when compared to a similarly viscous PVP solution, using planar flagellar beating patterns when swimming close to a wall [79]. Numerical models for microswimmers in viscoelastic fluid have not always agreed well with experimental data, yet various fluid models have shown that the elasticity of fluid plays a role in enhancing motility [84,85,86].

**Figure 6:**
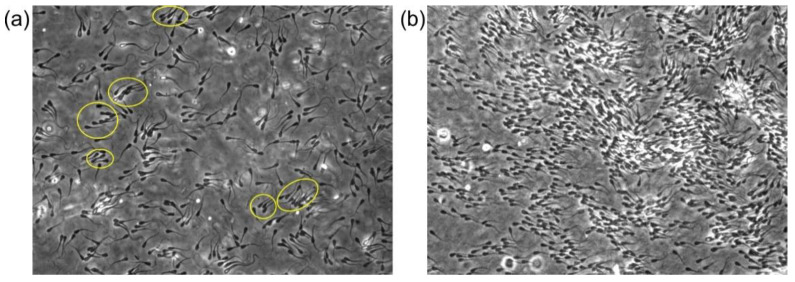
Bull sperm collective swimming in viscoelastic fluid. (**a**) At lower sperm numbers and 1% PAM solution, sperm form nonbinding clusters (yellow ovals) in which several neighboring sperm swim in the same direction. (**b**) At higher sperm numbers and 0.7% PAM plus 1% PVP solution (PVP was added to increase the fluid viscosity to reduce thermal-like randomization), after a pulse of flow was applied and had dissipated, a mass of sperm swam toward the same direction.

The viscoelasticity of the fluid not only affects the motility of individual sperm, but also provides additional ways for sperm to interact mechanically with each other. In MC solutions, it has been shown that flagellar beating of neighboring bovine sperm become synchronized [87]. Further, bovine sperm swim in clusters in highly elastic PAM solutions, while the orientations of the sperm within each cluster are roughly the same, as shown in Figure 6a [79]. The mechanism of how sperm interact with each other through the viscoelastic fluid is still under investigation to determine whether it can be seen as arising from the elasticity of the fluid [88] or as the result of the fluid flow [81]. More detailed fluid movement measurement, combined with hydrodynamic numerical simulations, will provide more evidence on the specific interaction mechanism. Due to the similarities in the morphologies of mammalian sperm species, it is possible that the collective behaviors of sperm in viscoelastic fluid exists widely [89].

## 4. Cooperative Movement of Sperm

The sperm clustering discussed in the previous section provides a mechanism for sperm to interact with each other through the surrounding fluid, and the interaction allows cooperation between sperm. In the semen of many mammalian species, sperm are highly concentrated in a viscoelastic seminal fluid. It was first observed in undiluted sheep semen that sperm swim in wave-like formations [90], which is also referred to as mass or massal motility. Interestingly, the quality of the massal motility in sheep semen was found to be correlated to the fertility of the male [91]. While the exact mechanism underlying this correlation is not known, the viscoelastic properties of human semen were also found to be correlated with the percentage of motile sperm and other motility parameters [92]. Indeed, the massal motility of sheep sperm can be analyzed using this framework [93]. In this context, through tuning the cell−cell interaction by adjusting the fluid viscoelasticity and the sperm cell density, sperm of other species, such as bovine, can form similar waves under the right conditions. Figure 6b is a snapshot of a high concentration of bovine sperm swimming in the same direction after aligning sperm momentarily using a pulse of flow, providing visualization of the sperm behavior analyzed in Ref. [93]. In theoretical physics, it has been known that, for self-propelled objects like sperm, local alignment with neighbors (such as those facilitated by the viscoelastic fluid) can lead to large-scale moving of objects in the same direction across a long distance [94,95]. Indeed, the massal motility of sheep sperm can be analyzed using this theoretical framework [93].

**Figure 7:**
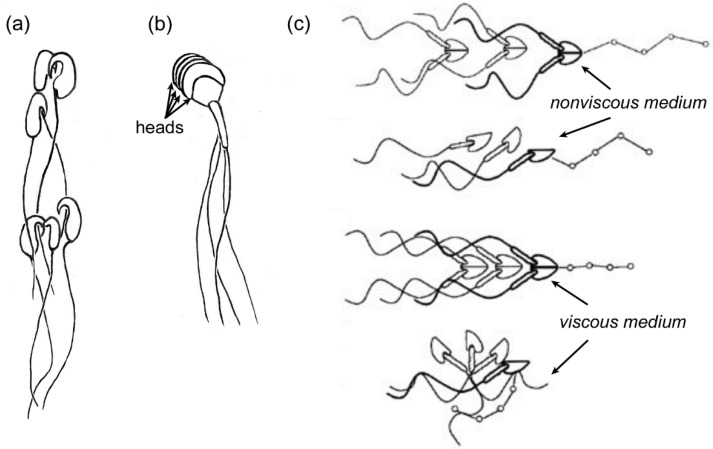
Illustrations of sperm aggregation in three species. (**a**) A small sperm train, in which sperm from wood mice (*Apodemus sylvaticus*) were joined when the hooks on their heads latched onto flagella or hooks of neighboring sperm. (**b**) Rouleaux in sperm from guinea pigs (*Cavia porcellus*), in which heads are stacked together. (**c**) Shows how sperm from the grey short-tailed opossum (*Monodelphis domestica*) form pairs and how single sperm and pairs move in non-viscous and viscous media. Paired sperm swim progressively in non-viscous and viscous media, while single sperm only swim progressively in non-viscous medium (opossum sperm illustrations used with permission from Ref. [105]. Copyright 1995 Oxford University Press.).

The cooperative movement of sperm can also be found with actual mechanical attachment of sperm to each other. In the wood mouse, *Apodemus sylvaticus*, sperm have hooks on their heads that, upon release of the sperm from the epididymis, latch onto the hooks or flagella of neighboring sperm, resulting in the formation of moving trains of cells (Figure 7a). Trains of 10–50 sperm were recovered from the uterus after mating [96]. When trains were allowed to form in vitro from freshly-obtained epididymal sperm, the swimming velocity of the trains was greater than that of solitary sperm [96]. It was proposed that such trains could have evolved as a result of sperm competition [96]. In support of this proposal, it was found that, in the deer mouse *Peromyscus maniculatus*, in which females mate with several males in a short time period, sperm aggregate preferentially in vitro with sperm from the same male over sperm from other males; whereas, sperm from the monogamous species, *Peromyscus polionotus,* aggregate indiscriminately with sperm from other males [97]. The mechanism of the tendency of sperm to aggregate preferentially with others from the same male is unknown, but proposed to be due to a homophilic adhesion protein [97].

Many species of muroid rodents, but not all, have hooks on their heads [98,99](see paper by Hook and Fisher in this issue). When 20 species with hooks were examined, few of them produced sperm that aggregated—at least in vitro [99]. This observation leads to the question of whether hooks serve other purposes in sperm, perhaps in physical interactions with the female tract. Interactions with female wall architecture, fluid flows in the tract, and fluid viscosity have yet to be studied in hook-headed sperm swimming separately or in aggregates.

Guinea pigs (*Cavia porcellus*), which are non-muroid rodents in the family Caviidae, produce sperm that stack together in “rouleaux” (Figure 7b) in the epididymis. That is, heads are adhered to each other via their broad surfaces. The rouleaux aggregations are maintained in the female tract, even into the oviduct [100,101,102]. Still, nothing is known about the mechanical interactions of sperm rouleaux with the female tract walls or fluids.

The sperm of new world marsupial mammals, the opossum species *Didelphis virginiana* [103] and *Monodelphis domestica* [104], adhere head-to-head in pairs. The structure formed by the pairs looks like a single biflagellate pusher microswimmer (Figure 7c). Pairing enhances the passage of these sperm through viscous media and thus may provide an advantage for sperm swimming in the viscous fluids of some regions of the female tract [105]. The pairs separate in the ampulla of the oviduct shortly before fertilization [103,106].

Altogether, various types of sperm aggregations have been observed in a broad array of mammalian species; however, maintenance of sperm aggregations in the female tract does not appear to be a common phenomenon. Our understanding of the mechanisms and functions of aggregation is still poor.

## 5. Apparent Co-Evolution of Sperm and Female Reproductive Tract Physical Traits

The physical cooperation of sperm with the female tract indicates that there has been co-evolution of the female and sperm physical traits. In this section, we will focus on representative physical interactions in areas of the female tract where they are best known. Nevertheless, there is evidence that physical cooperation exists throughout the female tract.

In humans and cattle (*Bos taurus*), there is an array of physical mechanisms that support the movement of sperm into and through the cervix. In these species, males deposit semen in the anterior vagina at the entrance to the cervix [30]. At insemination, sperm are densely packed in viscoelastic semen, which results in massal motility of the sperm [89]. As the masses of moving sperm contact the entrance to the cervix, they encounter infoldings of the cervical surface that radiate from the opening of the central canal [5,107]. Due to wall effects on pusher microswimmers, the parallel swimming sperm would tend to enter the infoldings with the same orientation. If sperm with random orientations were to approach the infoldings, it is possible that they would interfere with each other’s passage into the infoldings—especially at the high density of sperm in semen. Probably, the parallel swimming of sperm in the semen reduces the incidence of interference. After entering the infoldings, sperm are guided deeper into the cervix by the gentle downward flow of fluid that flows throughout the cervix. As the sperm continue into the cervix, they may encounter the many microgrooves that line the walls. These microgrooves have been traced through the length of the cervix into the main body of the uterus in cows [5].

**Figure 8:**
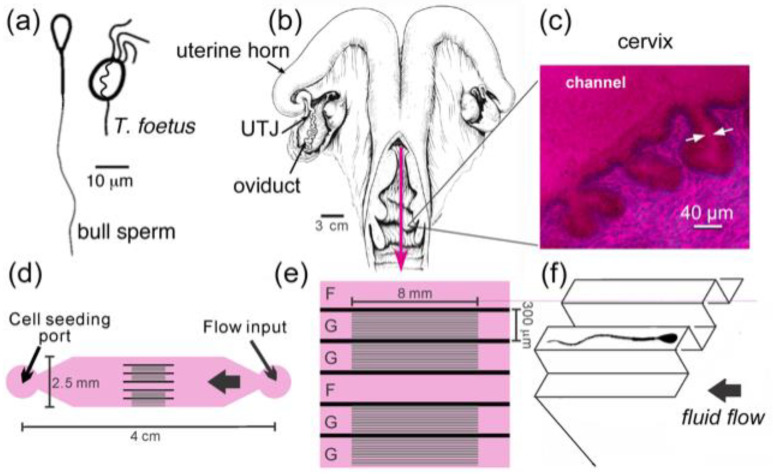
A microfluidic device designed to model fluid flows and microgrooves within the cervix. (**a**) Diagrams of bull sperm and *T. foetus*. (**b**) Illustration of a bovine female reproductive tract (from Ref. [108]). UTJ (uterotubal junction). The pink arrow points in the direction of fluid flow through the cervix. (**c**) Microgrooves are seen in PAS/hematoxylin-stained frozen sections of bovine cervix. (**d**) Diagram of microfluidic device that recreates the microgrooves and fluid flows of the bovine cervix. The sperm seeding port is on the left side and the flow inlet on the right; they are connected by channels with and without microgrooves. Detail of the channel design in the middle of the device: (**e**) *G* denotes channels with microgrooves in the upper surface and *F* denotes a control channel without grooves. (**f**) A 3D drawing illustrates the details of grooved channels. The cross-sectional dimensions of the microgrooves are 20 μm × 20 μm (drawing not to scale). Adapted with permission from Ref. [36]. Copyright 2015 Springer Nature.

In 1989, Mullins and Saacke [5] proposed that the microgrooves that run the length of the cervix in cows serve as “privileged paths” for sperm, protecting them from the main outflow in the center of the cervical canal, which carries spent leukocytes and cellular debris out from the uterus [5]. Our group further expanded and tested the “privileged paths” concept by building a microfluidic model of the microgrooves in the cervix (Figure 8) [52,53]. We first showed that sperm did swim upstream close to walls and in microgrooves in this model. Then, we tested another potential benefit of the microgrooves—in preventing upstream transit of flagellated pathogens while facilitating the upstream movement of sperm. For this test, we chose a common, motile, sexually transmitted bovine protozoan pathogen, *Tritrichomonas foetus*, and compared its ability to move upstream through the model with that of bull sperm. *T. foetus* is roughly the size of bull sperm, and it also swims using flagella, but it is not a pusher-type microswimmer. In contrast to the behavior exhibited by sperm in our microfluidic model, *T. foetus* did not swim along the walls or enter the microgrooves, and it was swept downstream by the gentle flows that caused sperm to swim upstream [53]. These results could indicate co-evolution of the cervix and sperm to promote sperm selection and guide sperm toward the site of fertilization, while protecting the uterus and upper tract from pathogens by flushing them down to the vagina.

The fluid in the cervical canal, infoldings, and microgroove passages is highly viscoelastic (more elastic than liquefied semen), due to the mucus secretions, but is readily penetrated by sperm of normal motility and morphology. In fact, cervical mucus has been shown to impede passage of abnormally shaped sperm, thereby playing a role in sperm selection [109,110,111,112]. The percentage of morphologically abnormal human sperm that had entered cervical mucus samples from women in estrus was <10%, compared with 25–75% abnormal sperm in the original semen samples. Sperm with abnormal flagella were rarely seen in the mucus [109]. These findings suggest the cervical mucus selects against morphologically abnormal sperm, particularly flagellar abnormalities. It has also been shown in humans that cervical mucus selects sperm with less DNA fragmentation [113]. Nevertheless, because actual cervical mucus was used in these studies, the effects of physical interactions could not be separated from the effects of molecular interactions between sperm and the components of cervical mucus. Now that rheological measurements can be made of mucus and molecularly inactive substitutes, a similar study could reveal the physical selective capacity of cervical mucus.

In cows and humans, the cervical mucus is only receptive to sperm penetration in the preovulatory estrous period when estrogen is the dominant sex steroid. After ovulation, when progesterone is the dominant sex steroid, the cervical mucus becomes less watery, more viscous and sticky, and blocks migration of sperm into the cervix [33]. It also serves as an antimicrobial barrier [114].

As the cervix is the junction between the vagina and uterus, the UTJ is the junction between the uterus and the oviduct. Consequently, some structural similarities exist between them, especially in species that deposit semen directly into the uterus. In the case of the pig, for example, a UTJ lies at the upper end of each of the two uterine horns. The UTJ projects fingerlike structures into the uterine cavity [115]. If sperm were to contact these structures, they would be led into valleys between longitudinal mucosal folds lining the UTJ. The valleys could lead sperm to the isthmus of the oviduct. In the estrous period in pigs, watery viscoelastic mucus fills the UTJ, as in the estrous bovine cervix. Altogether, watery viscoelastic mucus and tissue architecture may serve to select sperm and facilitate their migration up the female tract.

It has long been established that sperm must have normal motility in order to enter and pass through the UTJ [116]. Surprisingly, while investigating the functions of sperm-specific proteins in gene knockout (KO) mouse strains, it was found that a certain strain of KO sperm with normal morphology and motility were unable to pass through the UTJ [117]. Subsequently, the deletion of genes for a number of sperm-specific proteins resulted in the same infertile male phenotype [118]. The proteins produced by these genes were either plasma membrane proteins or were involved in bringing proteins to the plasma membrane. This seemed to indicate that sperm passage through the UTJ might involve some sort of molecular interaction between one or more sperm proteins and receptors lining the oviduct. However, recently, evidence has arisen to indicate that the proteins play a role in aggregating sperm in the uterus, and it has been proposed that the aggregated sperm, which are aligned in the same direction, are able to push their way into the UTJ, or push open the UTJ, which is something that single sperm seem unable to do [118]. This interesting discovery requires further investigation as a previously unknown function of sperm aggregation. This also highlights the significance of understanding the mechanical force generated by swimming sperm when they collide into structures, also known as swimming pressure in physics, particularly to compare the force generated by single and aggregated sperm.

Better imaging technology that allows the visualization of sperm in the female tract could answer a lot of questions, yet the challenges to such imaging include the light scattering and autofluorescence of tissue. One possible improvement may come from shortwave infrared fluorescence microscopy, as it has recently emerged as a method to achieve high temporal (>27 frames per sec) and spatial (sub-millimeter) resolution with better tissue penetration than near-IR. This technology could be used humanely on awake animals [119]. It remains to be explored how much this new technology will reveal.

## 6. Implications for Clinical Applications

In vitro fertilization (IVF), including intracytoplasmic sperm injection (ICSI), has become the treatment of choice for various kinds of human infertility; however, these procedures have been associated with heightened risk of low birth weight and preterm birth when compared with ovarian stimulation and natural conception [120,121]. To improve the outcome of IVF, the cooperation between the female tract and sperm could be used to design a selective in vitro environment similar to what occurs in vivo. The technology of the production of microfluidics devices provides opportunities to recreate the physical environment of the female tract on a microfluidics chip. Simulating cellular and molecular environments could also improve results. Nevertheless, much more information is needed about the environment of the female tract in order to create accurate microfluidic models. With regard to the physical environment, more information is needed about wall architecture, viscoelasticity, and the rate of fluid flows in the organs of the female reproductive tract. While scanning electron microscopy has revealed much about wall architecture, more needs to be learned about the physical properties of the walls in living tissue, such as the rigidity of surfaces and how the presence of motile cilia affects sperm interactions with walls when the sperm are not binding to the cilia. To the last point, the capability of culturing functioning epithelial layers in vitro would be greatly beneficial for understanding sperm−cilia interaction. Furthermore, little is known of the viscoelasticity of fluids in the tract, despite recent rheological measurements of cervical mucus. Lastly, more needs to be learned about the rate of fluid flow in various parts of the tract and how wall architecture affects local fluid flows.

Traditional methods of preparing the sperm of various species for IVF include (1) removal of seminal plasma by dilution and centrifugation of a semen sample, (2) removal of seminal plasma and selection of live sperm by density gradient centrifugation, and (3) collection of live sperm by allowing motile sperm to swim up out of whole semen or a centrifugation pellet of washed sperm [122]. These methods can expose the sperm for relatively long periods to temperature and pH fluctuations, as well as the physical stress of centrifugation [123]. Microfluidics can be used to minimize handling and the exposure of sperm to detrimental conditions by bypassing centrifugation and applying a semen sample directly to a microfluidic device, where sperm can be separated from semen and selected for the best motility and morphology [124].

A wide range of microfluidic devices have been proposed to perform semen/sperm separation and/or sperm selection for IVF ([125,126,127] for example, Ref. [128] for a review of the subject). It has been shown that some of the selection methods that are based on the physical environment reduce sperm DNA fragmentation in selected sperm [129,130], although the mechanism behind this correlation is not well understood. Much opportunity remains to incorporate the physical attributes of sperm interaction with the female tract into microfluidics devices to improve fertilization, and subsequent implantation and development successes [128].

## 7. Conclusions

Because the mammalian female reproductive tract serves several reproductive functions, it is exposed to a variety of selection pressures. This makes it difficult to identify the selection that leads to the co-evolution of sperm and the tract. So far, some evidence for co-evolution has been provided in exceptional genetic models, such as *Drosophila melanogaster* [8]. Nonetheless, clues about the co-evolution of physical traits have been uncovered in mammals, such as cervical microgrooves serving as privileged pathways for sperm in *Bos taurus.* These clues can be used to develop new microfluidics methods for the IVF treatment of infertility.
